# Deep learning and machine learning predictive models for neurological function after interventional embolization of intracranial aneurysms

**DOI:** 10.3389/fneur.2024.1321923

**Published:** 2024-01-24

**Authors:** Yan Peng, Yiren Wang, Zhongjian Wen, Hongli Xiang, Ling Guo, Lei Su, Yongcheng He, Haowen Pang, Ping Zhou, Xiang Zhan

**Affiliations:** ^1^Department of Interventional Medicine, The Affiliated Hospital of Southwest Medical University, Luzhou, China; ^2^School of Nursing, Southwest Medical University, Luzhou, China; ^3^Wound Healing Basic Research and Clinical Application Key Laboratory of Luzhou, Southwest Medical University, Luzhou, China; ^4^Department of Oncology, The Affiliated Hospital of Southwest Medical University, Luzhou, China; ^5^School of Medical Information and Engineering, Southwest Medical University, Luzhou, China; ^6^Department of Pharmacy, Sichuan Agriculture University, Chengdu, China; ^7^Department of Nursing, The Affiliated Hospital of Southwest Medical University, Luzhou, China; ^8^Department of Radiology, The Affiliated Hospital of Southwest Medical University, Luzhou, China

**Keywords:** radiomics, deep learning, machine learning, prediction model, artificial intelligence

## Abstract

**Objective:**

The objective of this study is to develop a model to predicts the postoperative Hunt-Hess grade in patients with intracranial aneurysms by integrating radiomics and deep learning technologies, using preoperative CTA imaging data. Thereby assisting clinical decision-making and improving the assessment and prognosis of postoperative neurological function.

**Methods:**

This retrospective study encompassed 101 patients who underwent aneurysm embolization surgery. 851 radiomic features were extracted from CTA images. 512 deep learning features are extracted from last layer of ResNet50 deep convolutional neural network model. The feature screening process pipeline encompassed intraclass correlation coefficient analysis, principal component analysis, *U* test, spearman correlation analysis, minimum redundancy maximum relevance algorithm and Lasso regression, to identify features most correlated with postoperative Hunt-Hess grading. In the model construction phase, three distinct models were constructed: radiomics feature-based model (RSM), deep learning feature-based model (DLM), and deep learning-radiomics feature fusion model (DLRSCM). The study also calculated the radiomics score and combined it with clinical data to construct a Nomogram for predictive modeling. DLM, RSM and DLRSCM model was constructed by 9 base algorithms and 1 ensemble learning algorithm – Stacking ensemble model. Model performance was evaluated based on the area under the Receiver Operating Characteristic (ROC) curve (AUC), Matthews Correlation Coefficient (MCC), calibration curves, and decision curves analysis.

**Results:**

5 significant radiomic feature and 4 significant deep learning features were obtained through the feature selection process. These features were utilized for model construction. Bootstrap resampling method was used for internal validation of the models. In terms of model evaluation, the DLM model, the stacking ensemble algorithm results achieved an AUC of 0.959 and MCC of 0.815. In the RSM model, the stacking ensemble model AUC was 0.935 and MCC was 0.793. The stacking ensemble model in DLRSCM outperformed others, with an AUC of 0.968 and MCC of 0.820. Results indicated that the ANN performed optimally among all base models, while the stacked ensemble learning model exhibited the highest predictive performance.

**Conclusion:**

This study demonstrates that the combination of radiomics and deep learning is an effective approach to predict the postoperative Hunt-Hess grade in patients with intracranial aneurysms. This holds significant value in the early identification of postoperative neurological complications and in enhancing clinical decision-making.

## 1 Introduction

Intracranial aneurysm (IA) is a prevalent and dangerous cerebrovascular disease characterized by the local dilation of cerebral arteries, which may lead to subarachnoid hemorrhage and other severe neurological complications, posing significant risks to patients (1, 2). Endovascular embolization of IAs is a widely used treatment method, involving the use of micro guidewires and catheters to deliver coils and other adjunct materials into the aneurysm sac, effectively occluding the aneurysm and preventing rebleeding (3, 4). However, the postoperative assessment of patients’ clinical neurological status and prognosis prediction remains challenging.

The Hunt–Hess grading system, which is widely employed for patients with IAs, allows for the description of clinical manifestations and the extent of neurological impairment, guiding treatment decisions and forecasting patient outcomes (5, 6). Hunt-Hess starting at grade 3 indicates that slightly focal neurologic deficits. Grade 5 is the most severe (Table 1). Clinicians want to predict before surgery whether a patient’s neurologic function will change from below grade 3 to grade 3 and above after surgery (7).

**Table 1:** Hunt-Hess grade scale (7).

Grade	Symptom
Grade I	Asymptomatic or mild headache and mild neck stiffness.
Grade II	Moderate to severe headache. Nuchal rigidity, no neurological deficit except for cranial nerve palsy.
Grade III	Drowsiness, confusion, or mild focal neurologic deficits.
Grade IV	Stupor, moderate to severe hemiparesis, possibly early decerebrate rigidity and vegetative disturbances.
Grade V	Deep coma, deregulation, near-death state

However, relevant predictive factors for postoperative Hunt–Hess grading in patients are lacking before the IA intervention. Currently, the assessment heavily relies on the clinical experience of healthcare provider to determine the extent of neurological impairment in patients following the procedure (8). However, the accuracy and objectivity of these assessments may be influenced by subjective factors, limiting the precision of patient prognosis evaluation. Therefore, seeking additional and more reliable objective predictive factors and methods for forecasting the prognosis of patients after IA endovascular embolization has become increasingly important.

In recent years, remarkable advancements have been witnessed in the field of medical imaging due to emerging technologies such as radiomics, machine learning, and artificial intelligence (9). Radiomics is a quantitative approach that extracts a large number of features from medical images, providing an objective reflection of various characteristics of pathological changes, including shape, size, texture, and so forth. This method offers a new perspective for disease diagnosis and prognosis (10). On the contrary, machine learning and artificial intelligence techniques can process and analyze vast amounts of medical imaging data, enabling the construction of sophisticated prediction models that can automate the prognostic assessment of patients and ultimately assist clinicians in making clinical decisions (11, 12).

**Figure 1:**
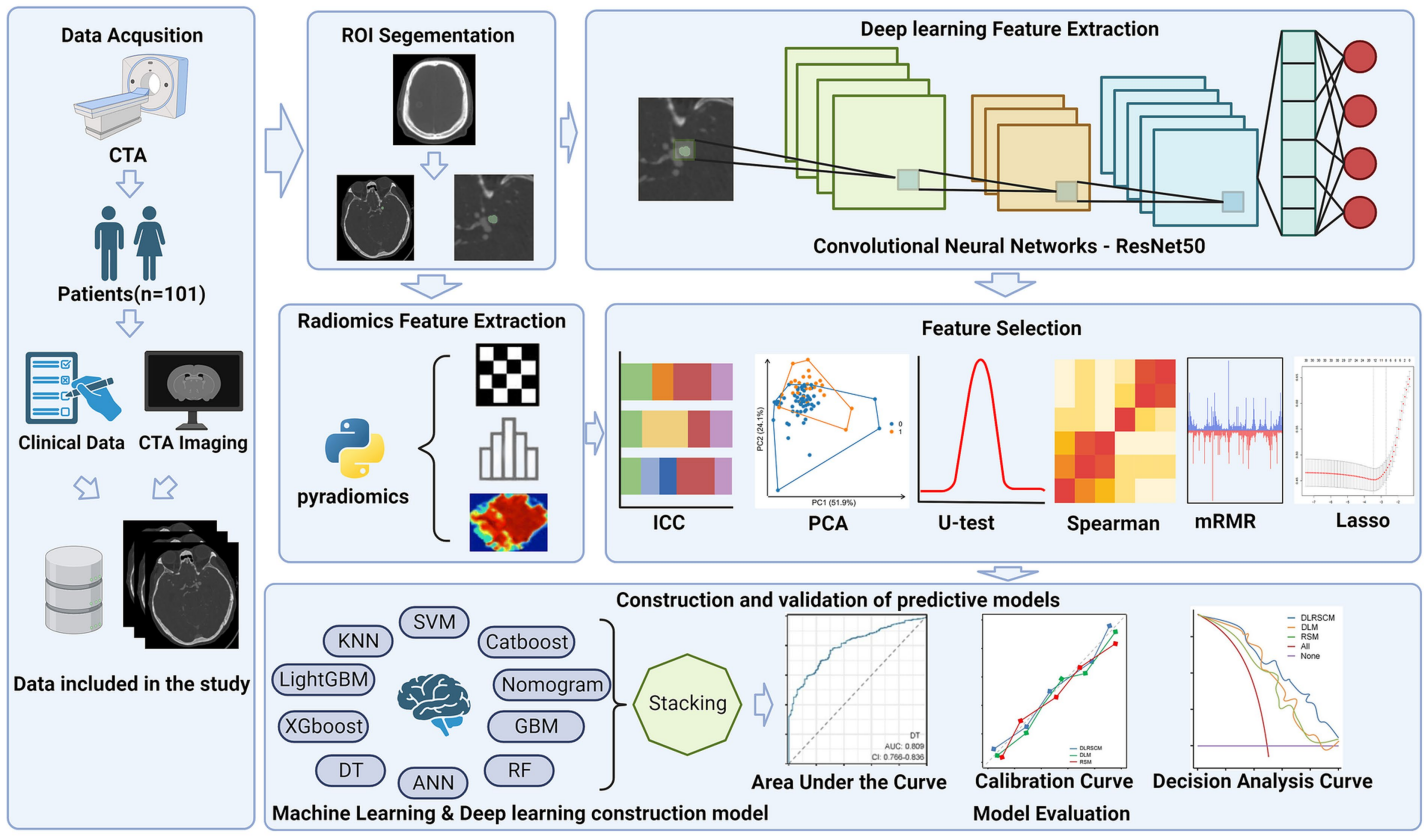
Flowchart of this study.

In this study, we extracted radiological and clinical features based on patients’ preoperative CT angiography (CTA) images and clinical data using machine learning and deep learning methods. Next, we constructed 32 prediction models using various machine learning and deep learning methods. The study aimed to construct three distinct models to investigate their predictive abilities for the research objective. These models included Radiomics feature-based model (RSM), Deep learning feature-based model (DLM), and Deep learning-radiomics feature fusion model (DLRSCM). The RSM primarily relied on the radiomics features extracted from medical imaging data. Radiomics features provided quantitative information about the imaging characteristics of the tumor. The model employed various machine learning algorithms or statistical techniques to predict the postoperative Hunt–Hess classification. The DLM focused on using deep learning techniques to directly analyze the extracted features from medical imaging data. Deep learning algorithms, such as convolutional neural networks (CNNs), were used to learn and identify intricate patterns and features from the imaging data, which could further enhance the predictive performance of the model (13, 14). DLRSCM was a novel approach that aimed to integrate the strengths of radiomics and deep learning (15). By combining the radiomics features with deep learning, this model sought to leverage the complementary information from both sources, potentially leading to a more robust and accurate predictive model. By constructing and evaluating these three types of models, we intended to provide comprehensive insights into their respective advantages and limitations in predicting the postoperative Hunt–Hess classification for patients with IAs. The flowchart of this study is shown in Figure 1.

## 2 Materials and methods

### 2.1 Patients

This retrospective study included 101 patients with IA who were surgically treated at the Affiliated Hospital of Southwest Medical University from January 2019 to January 2022. The inclusion criteria were as follows: (1) patients with IA confirmed by Digital subtraction angiography (DSA); (2) patients with complete preoperative medical records and imaging data; and (3) patients who received standard treatment, that is, patients treated by surgical embolization. The exclusion criteria were as follows: (1) patients who had only a CT examination without DSA to confirm the diagnosis; (2) patients without a complete medical history; (3) patients with incomplete image data and image artifacts; and (4) patients who were not treated surgically. This study was conducted in strict accordance with the Declaration of Helsinki and approved by the Medical Ethics Committee of the Affiliated Hospital of Southwest Medical University (No. KY2023041). The requirement for obtaining informed consent from the patients was waived due to the retrospective nature of this study.

### 2.2 CT image acquisition and preprocessing

All images were obtained using Philips IQon spectral CT (Philips, Netherlands). The scanning mode was as follows: spiral scanning, with scanning direction from the side of the foot to the side of the head. The scanning parameters were as follows: 100–120 kVp, automatic milliampere-second technique, x-ray tube rotation time 0.33 s/round, pitch 1.046, Field of view (FOV) (200–250) mm × (200–250) mm, window width 600 HU, window level 300 HU, layer thickness 0.90 mm, interval 0.90 mm, and detector width 8 cm. The automatic contrast tracking trigger used a scanning technique, monitoring the level of the aortic arch, trigger threshold 80–120 HU, and standard reconstruction algorithm Standard/B30. All captured images were saved in digital imaging and communication in medicine (DICOM) format.

### 2.3 Region of interest segment and radiomics feature extraction

All images were taken from a DICOM-format picture archiving and communication system and transferred to 3D Slicer (version 4.21). 3 neuroimaging physicians with ≥10 years of clinical experience in radiology used the 3D Slicer software to manually segment regions of interest (ROIs) on the CT images and perform radiomics feature extraction on the outlined ROIs. Radiomics allows for the conversion of medical images into high-dimensional, quantifiable data. This process involves extracting a large number of features from ROIs, which include aspects like shape, texture, intensity, and volume (16). These features provide a detailed and quantifiable description of the IA area.

### 2.4 Deep learning feature extraction using ResNet50

**Figure 2:**
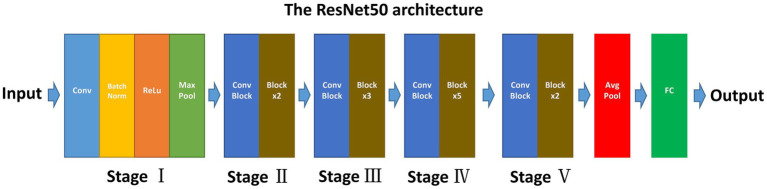
Schematic diagram of ResNet50 structure. ResNet50 consisted of 50 layers and 5 stages.

The ResNet50, known for its deep convolutional neural network (CNN) architecture, offers several advantages for medical image analysis. Its design addresses common challenges associated with deep learning models, such as the vanishing gradient problem, making it suitable for extracting nuanced features from complex image data (17). ResNet50 comprises 50 layers, including convolutional layers, pooling layers, and fully connected layers (Figure 2) (18). This depth allows it to learn complex patterns in image data and its adaptability to various types of image analysis tasks make it an ideal choice for our study.

#### 2.4.1 Pre-training and fine-tuning

**Figure 3:**
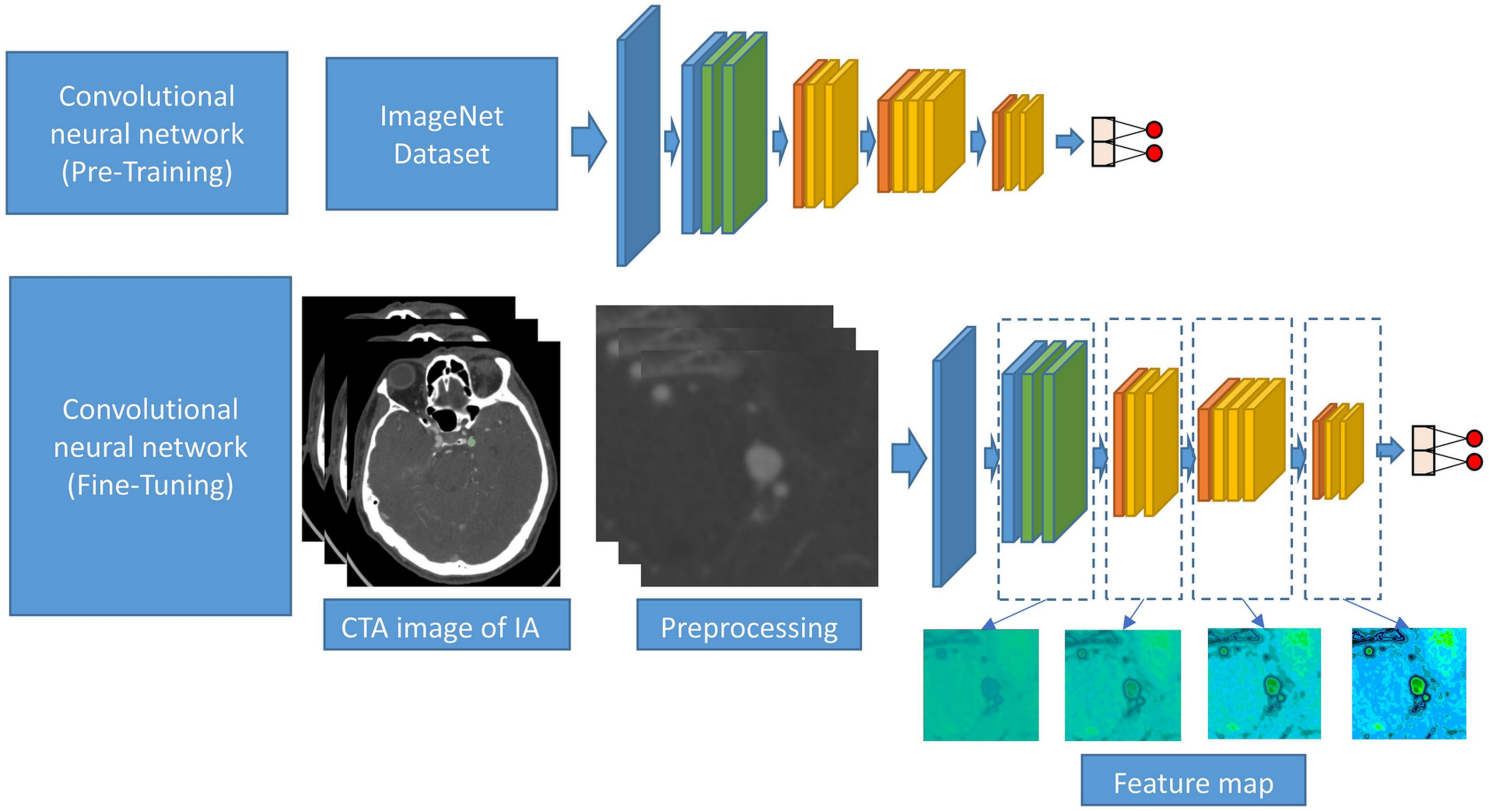
Schematic diagram of the CNN procession of this study. The CNN was pre-trained using ImageNet and fine-tuned using the CTA image data of IA.

This study approach leverages a ResNet50 model pre-trained on ImageNet, a comprehensive visual database. This pre-training imparts the model with a foundational understanding of a wide range of visual features, which is crucial for initial feature recognition. The fine-tuning process, tailored to our study’s requirements, adapts this model to the unique characteristics of CTA images specific to intracranial aneurysms (Figure 3). This step is critical as it aligns the model’s learning focus with the specific textures, shapes, and patterns relevant to aneurysms, enhancing the model’s accuracy and specificity in identifying relevant features in our dataset.

#### 2.4.2 Feature extraction from network

The primary role of the ResNet50 model is the extraction of deep learning features from last layer of the network. This approach is chosen for its ability to capture high-level, abstract representations of the data, which are crucial in medical imaging analysis, especially for identifying complex patterns within cerebral vascular structures (19). The last layer in ResNet50 consolidates the information processed through all preceding layers, providing a comprehensive and detailed set of features. These high-level features are vital for accurately characterizing intracranial aneurysms, as they encompass the most informative and discriminative aspects of the CTA images, refined through the model’s depth and complexity. Additionally, by extracting features from the final layer, we ensure that the learned representations are specific to the medical imaging domain, having been fine-tuned on our dataset. This specificity is key to achieving high accuracy in identifying and assessing cerebral aneurysms, making it a strategic choice for our study’s objectives in neuroimaging analysis.

### 2.5 Deep learning feature and radiomics feature screening

**Figure 4:**
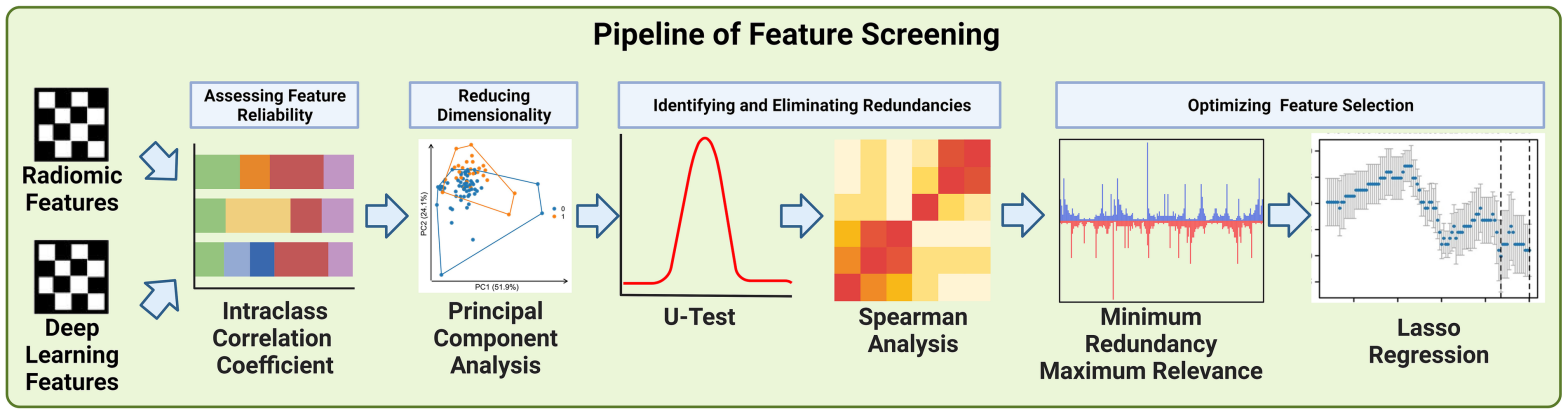
Schematic of feature screening pipeline.

Due to the large number of radiomics features and deep learning features extracted, we developed a pipeline to filter the important features step by step, the steps of feature filtering are shown in Figure 4.

#### 2.5.1 Intraclass correlation coefficient

The Intraclass Correlation Coefficient (ICC) played a pivotal role in the screening of deep learning and radiomics features in our study, serving as a key statistical tool to assess the reliability and consistency of measurements across different observers (20). In our context, ICC was utilized to evaluate how consistently neuroimaging physicians extracted features from the CT images, which is critical to ensure the integrity of our data analysis. The ICC is calculated using the formula:


$$ICC=\frac{MS_{B}-MS_{w}}{MS_{B}+\left(k-1\right)MS_{w}}$$

Where $MS_{B}$ is the mean square between neuroimaging physicians extracted features from the CT images, $MS_{w}$ is the mean square within neuroimaging physicians extracted features from the CT images, and k is the number of neuroimaging physicians.

A high ICC value, typically above 0.75, indicates a strong agreement among the observers, signifying that the features extracted are consistent and reliable, irrespective of the observer (21). This threshold was chosen to ensure the high quality and reproducibility of our feature dataset. Features failing to meet this threshold were considered unreliable and were excluded from further analysis. This step was crucial for maintaining the reproducibility, particularly important in studies involving multiple observers. By employing ICC in the initial phase of feature screening, we established a robust foundation for the subsequent, more complex phases of our analysis.

#### 2.5.2 Principal component analysis

Principal Component Analysis (PCA) was employed primarily to reduce the number of features derived from both deep learning and radiomics extraction methods. Given the large volume of features generated. The dimensionality of the data posed significant challenges in terms of computational efficiency and potential overfitting in subsequent analyses. PCA served as a crucial statistical technique to address these challenges. Its primary purpose was to condense the high-dimensional feature space into a lower-dimensional space that retains most of the original data variance (22).

The core process of PCA involves the computation of the covariance matrix of the data and its eigenvalue decomposition:


$$\sum =\frac{1}{n-1}\times {\left(X-\bar{X}\right)}^{T}\times \left(X-\bar{X}\right)$$

Where X represents the data matrix, and $\bar{X}$ is the mean vector. The covariance matrix Σ captures the variance and covariance between different features.

The subsequent eigenvalue decomposition is expressed as:


Σ×V=λ×V

This step involves finding the eigenvectors V and eigenvalues of the covariance matrix. The eigenvectors define the new feature space, and the eigenvalues indicate the variance captured by each eigenvector.

By transforming the data using these principal components, allowed us to maintain the integrity and informational value of the original features while significantly reducing their number. By doing so, we enhanced the manageability of our data.

#### 2.5.3 *U* test and spearman analysis

We utilized the Mann–Whitney U Test and Spearman’s rank correlation analysis to refine our selection of features derived from deep learning and radiomics techniques. The *U* Test, a non-parametric statistical method, was employed to determine the significance of each feature by comparing their distributions between different groups within our study cohort (23). This helped in identifying features that were statistically significant in distinguishing between the groups, such as patients with varying outcomes or characteristics. The U statistic is calculated as:


$$U=n_{1}n_{2}+\frac{n_{1}\left(n_{1}+1\right)}{2}-R_{1}$$

Where $n_{1}$ and $n_{2}$ are the sample sizes, and $R_{1}$ is the sum of the ranks in the first sample. The U statistic assesses whether one sample tends to have higher values than the other.

Concurrently, we used Spearman’s rank correlation analysis to assess the relationships between features. This analysis was crucial in identifying highly correlated features, indicating redundancy. Features that were strongly correlated with others were considered for removal since they provided similar information, thereby simplifying our feature set (24). Spearman’s correlation (ρ) assesses the relationship between two ranked features and is calculated as:


$$\rho =1-\frac{6\times \sum d_{i}^{2}}{n\left(n^{2}-1\right)}$$

Here, $d_{i}$ is the difference between the ranks of corresponding values of the two features, and n is the number of observations. This coefficient indicates the degree of correlation between the ranks of the features.

The integration of these two statistical methods – the *U* Test for determining feature significance and Spearman’s analysis for identifying feature redundancy – was instrumental in ensuring that the final set of features was both relevant and concise, enhancing the effectiveness and efficiency of our subsequent predictive modeling.

#### 2.5.4 Minimum redundancy maximum relevance and Lasso regression

The integration of the Minimum Redundancy Maximum Relevance (mRMR) algorithm and Lasso regression played a crucial role in our feature selection process. Initially, the mRMR algorithm was used to filter out features, ensuring that those selected were highly relevant to our predictive models while minimizing redundancy (25). This step was vital in balancing the inclusion of essential information and avoiding overlap among features. Following this, Lasso regression was applied to further refine the feature set. A key attribute of Lasso regression is its ability to perform feature selection and regularization simultaneously. This is particularly effective in high-dimensional data sets like ours. Lasso achieves this by imposing a penalty on the absolute size of the coefficients, which leads to some coefficients being exactly zero. Thus, features with non-zero coefficients are selected, while others are effectively excluded from the model. This process not only reduces the number of features but also assigns a non-zero coefficient to each selected feature (26). The Lasso regression can be expressed as:


$$\arg min_{\beta }=\frac{1}{2n}{\left|\left|y-X\beta \right|\right|}_{2}^{2}+\lambda {\left|\left|\beta \right|\right|}_{1}$$

Where ${\left|\left|y-X\beta \right|\right|}_{2}^{2}$ is the least squares term, λ is a tuning parameter that controls the strength of the penalty, β represents the coefficients, and ${\left|\left|\beta \right|\right|}_{1}$ is the L1 norm of the coefficients. The L1 penalty encourages sparsity in the coefficients, leading to some coefficients being shrunk to zero, thus effectively selecting a subset of features.

By combining mRMR for initial feature reduction and Lasso regression for determining the final set of significant features with their corresponding non-zero coefficients, we were able to create an optimally concise and informative feature set.

### 2.6 Construction of prediction models

#### 2.6.1 Phase I-basic model construction

The remaining deep learning features and radiomics features after screening will be used to construct radiomics feature-based model (RSM), deep learning feature-based model (DLM), and deep learning-radiomics feature fusion model (DLRSCM). The construction of each type of base model we performed by 9 supervised learning algorithms which include random forest (RF) algorithm, support vector machine (SVM) algorithm, gradient boosting machine (GBM) algorithm, CatBooost, artificial neural network (ANN) algorithm, XGBoost, LightGBM, decision tree (DT), and K-nearest neighbor (KNN). These nine algorithms are classical supervised learning algorithms that have been applied in many studies of medical predictive modeling (27). Each base model is trained independently, and their predictions are recorded. We denote the predictions from the k -*th* model for the i -*th* sample as $p_{ik\text{.}}$

#### 2.6.2 Phase II-stacking ensemble model construction

The second phase of the construction involved the development of a meta-model, also known as the secondary learner. This meta-model was learned from the predictions made by the basic models in the first phase. A new dataset is created for training the meta-model, where the features are the predictions from the base models. If there are N samples and K base models, the new dataset D for the meta-model will be:


$$D=\left\{\left(p_{1k},p_{2k},\ldots \ldots .,p_{Nk}\right)|k=1,2,\ldots ,K\right\}$$

The meta-model is then trained on this new dataset. The output of the meta-model is the final prediction, which can be denoted as:


$$\hat{y}=f\left(p_{1},p_{2},\ldots \ldots .,p_{K}\right)$$

Where $\hat{y}$ is the predicted output, $p_{K}$ are the predictions from the k -*th* base model, and f represents the learning function of the meta-model.

The meta-model effectively synthesized the insights gained from all the basic models. We selected the best-performing model from the first phase as the basis for our meta-model, ensuring that the most accurate and reliable predictive patterns were carried forward into the final ensemble model. The final Stacking Ensemble Model represented an integration of the diverse predictive capabilities of the individual basic models, channeled through the refined lens of the meta-model (28). The primary advantage of this stacking approach lies in its ability to synthesize the strengths and compensate for the weaknesses of individual models, leading to a more accurate and robust predictive tool. This method effectively integrates diverse predictive insights from various models, ensuring a comprehensive analysis (29). The best-performing model from the basic models was chosen as the secondary learner, ensuring that the final ensemble model capitalizes on the most effective predictive patterns.

### 2.7 Construction and evaluation of radiomics nomogram

Non-zero coefficients for radiomics features screened by Lasso regression were used for constructing the radiomics score (Rad-Score). The general formula for calculating the Rad-Score can be expressed as follows:


$$\mathrm{Radiomics}\;\mathrm{Score}=\overset{n}{\underset{i=1}{\sum }}\left(\beta_{i}\times X_{i}\right)$$

Where n represents the number of radiomics features selected by Lasso regression. $\beta_{i}$ is the non-zero coefficient assigned to the i -*th* radiomics feature by Lasso regression. $X_{i}$ is the value of the i -*th* radiomics feature for a given patient.

A nomogram was constructed using the Rad-Score in combination with previously analyzed statistically significant clinical data. The nomogram was designed to visually represent the contribution of each feature towards the predicted outcome. Each feature was assigned a score on the nomogram, with the total score correlating to a probability of a postoperative Hunt–Hess grading.

## 3 Experiments

### 3.1 Data and implementation details

#### 3.1.1 Baseline information analysis

A total of 104 patients with intracranial aneurysms data were included in this study. The continuous variables were analyzed using the mean standard deviation (Mean ± Standard Deviation) and Mann–Whitney U Test. Chi-square test, Yates correction, and Fisher’s exact probability were used to analyze in categorical variables. Typically, two-sided *p* values <0.05 indicated statistically significant differences.

#### 3.1.2 Radiomics feature extraction

In our study, radiomics feature extraction was carried out using the pyradiomics package, encompassing comprehensive image preprocessing and subsequent extraction of a wide array of radiomics features. The preprocessing phase involved normalizing the gray values of images, resampling voxel sizes to a standardized volume of 1 mm × 1 mm × 1 mm, and discretizing image gray values with a bin width of 25. Various image types were included in the analysis, such as original images, Gaussian-filtered images applying Laplacian of Gaussian functions with five different sigma values (1.0, 1.5, 2.0, 2.5, 3.0), and wavelet-filtered images. The feature extraction process covered an extensive range of radiomics features, including morphological, first-order, Gray Level Co-occurrence Matrix (GLCM), Gray Level Run Length Matrix (GLRLM), Gray Level Size Zone Matrix (GLSZM), and Gray Level Dependence Matrix (GLDM) features. In total, 851 radiomics features were extracted and all underwent z-score standardization for normalization.

#### 3.1.3 Deep learning feature extraction

The input CTA image size was 224 × 224 pixels of the aneurysm region segmented by a neuroimaging physician. Also, we used a pre-trained ResNet50 model on ImageNet. The model was fine-tuned using our CTA image data to extract deep learning features specific to vascular structures. For this study’s binary classification task, adjustments were made by modifying the size of the fully connected layer from 1,000 to 2. The image flip rotation was used to increase the size of the dataset. We applied the adaptive moment estimation optimizer with a learning rate of 10^−4^, weight decay 10^−5^ for 1,000 epochs using a batch size of 16. We fed the preprocessed CTA images into the ResNet50 model and extracted deep learning feature from the last layer.

#### 3.1.4 Feature screening

All features were normalized to ensure a standardized baseline for comparison. The Intraclass Correlation Coefficients (ICCs) were then used as confidence coefficients to assess interobserver and test–retest reliability. We set a threshold of ICC > 0.75 as the benchmark for favorable reliability and validity, a standard practice in studies where reproducibility is paramount (30). To further refine the feature set, the Mann–Whitney U test was applied to each feature to identify and eliminate redundancies. *p* value threshold of 0.05 was set for this purpose, based on conventional statistical standards that balance the need for sensitivity in detecting meaningful differences while controlling for false positives (31). This threshold was chosen to effectively identify features with strong discriminative ability, essential for the predictive accuracy of our models. Spearman correlation analysis also conducted to address the dependency between features. In cases where the correlation coefficient between two features exceeded 0.9, indicating high redundancy, one of the features was excluded. This threshold of 0.9 was selected to ensure a significant level of correlation that could imply redundancy, hence optimizing the feature set for diversity and informativeness (32). For further refinement, the Minimum Redundancy Maximum Relevance (mRMR) algorithm was utilized, providing an additional layer of filtering to enhance the relevance and minimize redundancy among the features. Finally, the Least Absolute Shrinkage and Selection Operator (Lasso) regression model was employed for dimensionality reduction. We optimized the penalty parameters using 7-fold cross-validation, specifically selecting the lambda. Min that corresponded to the lowest error. This approach ensured that the dimensionality reduction did not compromise the capability of the Lasso regression model (33).

#### 3.1.5 Training of prediction model

Bayesian optimization algorithm to determine the hyperparameter settings for all base models, ensuring optimal model performance. The parameters for Bayesian optimization were configured as follows: The initial number of random search steps was set to 5, providing a diverse starting point for the algorithm. This was followed by 50 iterations of Bayesian optimization to precisely adjust and optimize the hyperparameters. This approach effectively balanced exploration and exploitation, ensuring a comprehensive and efficient search of the hyperparameter space. The hyperparameter settings for each model determined by the Bayesian optimization algorithm are detailed in Supplementary material S1. Given the relatively small sample size of our study, traditional data splitting and cross-validation methods might not provide robust validation results (34). Therefore, we chose the Bootstrap resampling method as the internal validation approach for both the base models and the Stacking ensemble model. Specifically, we conducted 1,000 Bootstrap resampling, a parameter setting aimed at ensuring the sufficiency and robustness of model validation. This resampling method allowed us to more accurately assess the performance of the models while considering potential sample variability, thus ensuring the reliability and effectiveness of the models. This method is particularly suitable for situations with a small sample size, as it provides in-depth insights into the stability and generalizability of the models.

### 3.2 Evaluation measure

The performance of basic and stacking predictive models will be evaluated using two key metrics: the area under the Receiver Operating Characteristic (ROC) Curve (AUC) and the Matthews Correlation Coefficient (MCC). The AUC is a crucial measure in binary classification tasks, quantifying a model’s ability to discriminate between classes (35). The MCC, on the other hand, provides a balanced evaluation of the classification performance, especially useful in datasets with imbalanced class distributions. It takes into account true and false positives and negatives, offering a comprehensive measure of the model’s accuracy (36). The MCC is calculated using the formula:


$$MCC=\frac{\left(TP\times TN-FP\times FN\right)}{\sqrt{[}\left(TP+FP\right)\times \left(TP+FN\right)\times \left(TN+FP\right)\times \left(TN+FN\right)}$$

Where TP is the number of true positives, TN is the number of true negatives, FP is the number of false positives, and FN is the number of false negatives.

### 3.3 Experimental condition

The experimental environment is based on AMD EPYC 9754 2.25 GHz CPU, 128 GB RAM, over CUDA 11.8 and NVIDIA RTX 4090 24 GB GPU under a 64-bit windows system. Python’s PyTorch framework was used for the deep learning task of this study. The Scikit-learn library was used for the machine learning task of this study.

### 3.4 Experimental results

#### 3.4.1 Patient baseline analysis

Table 2 demonstrates the baseline characteristics of all patients in this study. The study included 101 patients with ruptured IAs treated by aneurysm embolization. The number of patients with postoperative Hunt–Hess grading <3 (*n* = 71) was higher than the number of those with postoperative grading ≥3 (*n* = 30). Preoperative Hunt–Hess grading was significantly correlated with postoperative Hunt–Hess grading (*p* < 0.05). Additionally, no statistically significant differences were found between the other variables (*p*>0.05).

**Table 2:** Clinical baseline information.

Characteristics	Hunt-Hess grade<3	Hunt-Hess grade ≥ 3	*p* value
*n*	71	30	
Gender, *n* (%)			0.463
Male	23 (22.8%)	12 (11.9%)	
Female	48 (47.5%)	18 (17.8%)	
Age, mean ± sd	54.662 ± 11.913	59.267 ± 12.194	0.081
Hypertension, *n* (%)			0.447
No	39 (38.6%)	14 (13.9%)	
Yes	32 (31.7%)	16 (15.8%)	
Diabetes, *n* (%)			0.435
No	70 (69.3%)	28 (27.7%)	
Yes	1 (1%)	2 (2%)	
Smoking, *n* (%)			0.871
No	64 (63.4%)	26 (25.7%)	
Yes	7 (6.9%)	4 (4%)	
Family History, *n* (%)			0.435
No	70 (69.3%)	28 (27.7%)	
Yes	1 (1%)	2 (2%)	
Stent Assist, *n* (%)			0.948
Yes	35 (34.7%)	15 (14.9%)	
No	36 (35.6%)	15 (14.9%)	
Aneurysm size, *n* (%)			0.571
≤5 mm	37 (36.6%)	13 (12.9%)	
5 mm-15 mm	30 (29.7%)	16 (15.8%)	
>15 mm	4 (4%)	1 (1%)	
Aneurysm location, *n* (%)			0.070
ACA	24 (23.8%)	6 (5.9%)	
MCA	25 (24.8%)	18 (17.8%)	
PCA	22 (21.8%)	6 (5.9%)	
Raymond grading, *n* (%)			1.000
1	68 (67.3%)	29 (28.7%)	
2	3 (3%)	1 (1%)	
Preoperative hunt-hess grading, *n* (%)			0.002
<3	62 (61.4%)	18 (17.8%)	
≥3	9 (8.9%)	12 (11.9%)	

The data in parentheses are percentages. *p* values were obtained after univariate analysis between each variable and postoperative Hunt–Hess grading.

#### 3.4.2 Radiomics and deep learning feature screening

**Figure 5:**
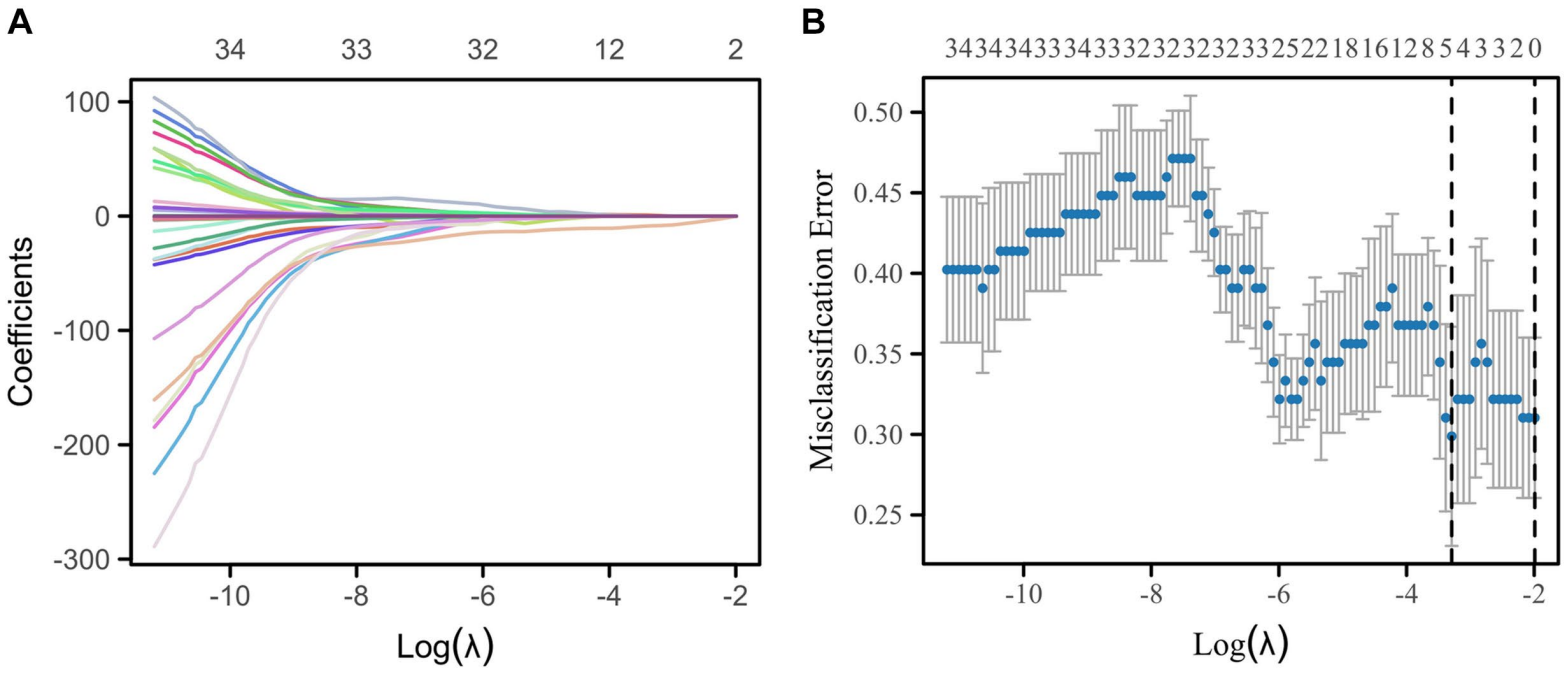
Lasso regression for the selection of radiomics features by 7-fold cross-validation and lambda.min selection of important features. **(A)** Variable trajectories of Lasso regression. **(B)** Lasso regression coefficient screening.

In this study, a total of 851 radiomics features and 512 deep learning features were extracted. The interobserver reproducibility of the feature extraction process was found to be favorable. Specifically, the reproducibility range for radiomics features was between 0.766 and 0.941, and for deep learning features, it was between 0.759 and 0.953. After dimensionality reduction using PCA, we obtained 267 radiomics features and 74 deep learning features. Redundant features were eliminated using the *U* test and Spearman correlation coefficient analysis, resulting in a final count of 136 radiomics features and 11 deep learning features. Subsequently, we used the mRMR algorithm to further select the most significant radiomics and deep learning features that were relevant to the postoperative Hunt-Hess grading. The mRMR output 39 radiomics features and 4 deep learning features. Given the still considerable number of remaining radiomics features, these were further subjected to Lasso regression analysis. The 4 deep learning features obtained were used for the construction of the DLM and were not included in the Lasso regression due to their already optimized number ensuring model efficiency. From the 39 radiomics features, Lasso regression ultimately retained 5 significant features (Figure 5). The titles of the five important features and their corresponding non-zero coefficients are shown in Table 3.

**Table 3:** Five radiomics features screened by Lasso regression and their corresponding nonzero coefficients.

Radiomics feature	Coefficient
original.firstorder.RootMeanSquared	0.000922077313959615
original.glcm.JointAverage	0.0785993197007064
wavelet-LHL.glcm.Idm	0.701727629619912
wavelet-LLH.glszm.SmallAreaLowGrayLevelEmphasis	−8.4144084384922
wavelet-HHH.glcm.ClusterShade	−0.0361369923060118

#### 3.4.3 Evaluation of multi-machine learning prediction model

**Figure 6:**
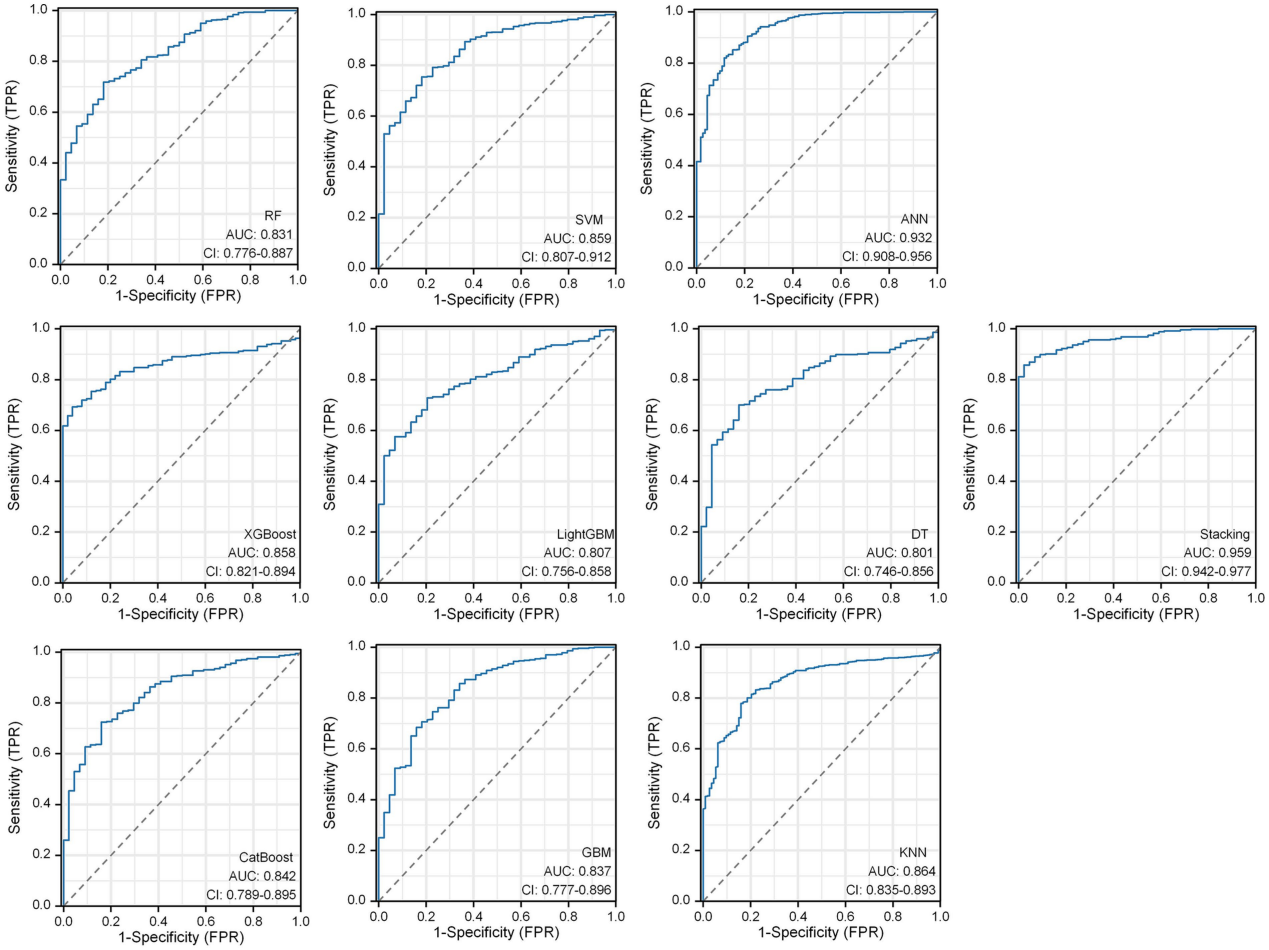
Predictive ability of the 10 DLMs evaluated using the AUC of the ROC curve.

**Figure 7:**
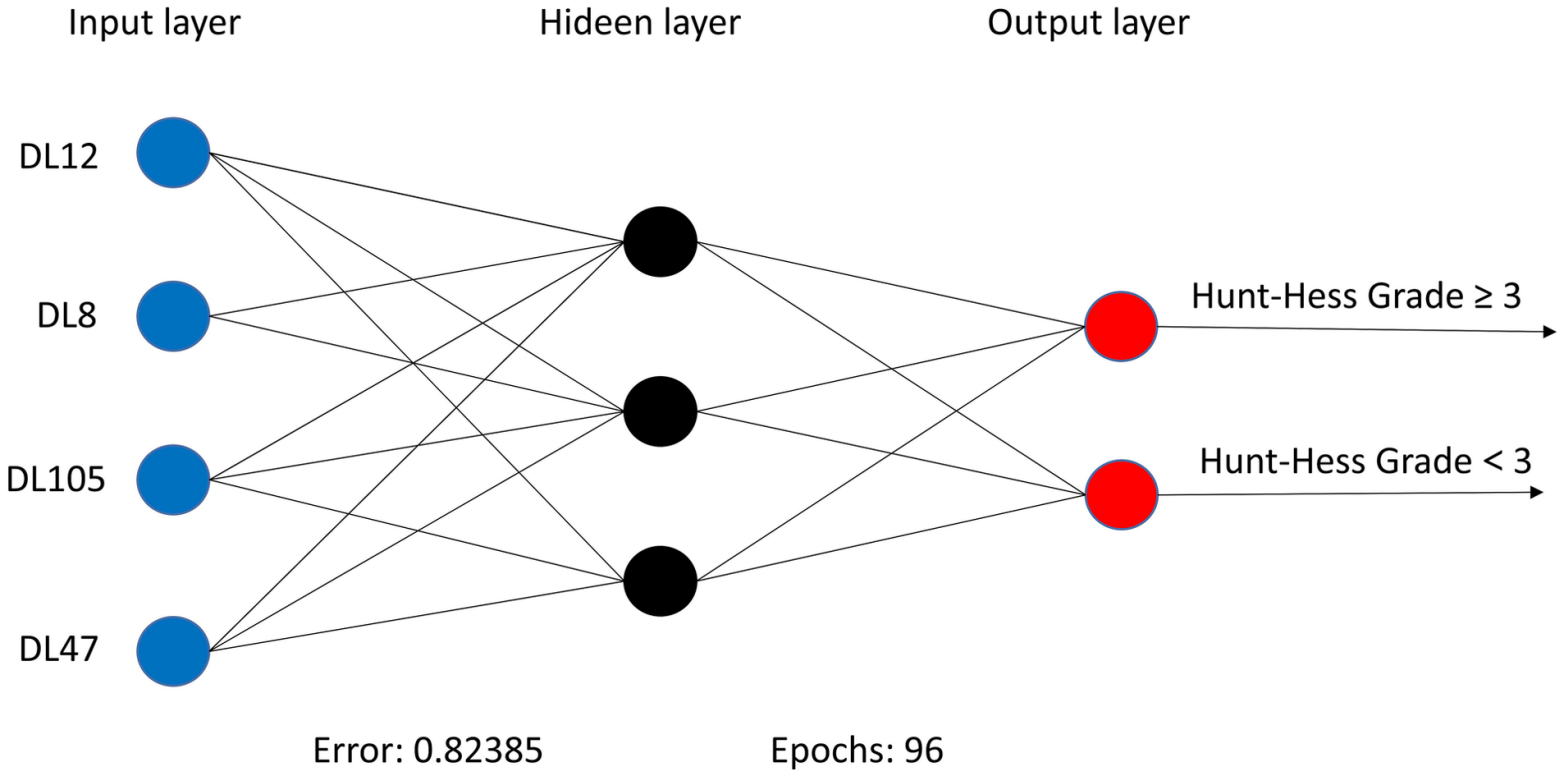
ANN algorithms for constructing DLM visualization, consisting of 4 input layers, 3 hidden layers, and 2 output layers.

Within the DLMs, the Artificial Neural Network (ANN) model emerged as the top performer, achieving an AUC of 0.932 and an MCC of 0.830 (Table 4 and Figure 6). The ANN model used to construct the DLM was trained with 96 epochs to reach a minimum error rate of 0.82385 (Figure 7). This outcome of ANN highlighted robust capability in analyzing complex data patterns. In contrast, models like the Decision Tree (DT) and LightGBM demonstrated lower performance, with AUCs of 0.801 and 0.807, respectively. Such disparities in performance among different models underscore the importance of careful model selection based on the specific nature of the data.

**Table 4:** Predictive ability of the 10 DLMs evaluated using the AUC and MCC.

Model	AUC	MCC
RF	0.831 (95%CI:0.776–0.887)	0.714 (95%CI:0.685–0.736)
SVM	0.859 (95%CI:0.807–0.912)	0.722 (95%CI:0.680–0.742)
ANN	0.932 (95%CI:0.908–0.956)	0.830 (95%CI:0.798–0.864)
XGBoost	0.858 (95%CI:0.821–0.894)	0.716 (95%CI:0.686–0.727)
LightGBM	0.807 (95%CI:0.756–0.858)	0.691 (95%CI:0.621–0.740)
DT	0.801 (95%CI:0.746–0.856)	0.638 (95%CI:0.602–0.686)
CatBoost	0.842 (95%CI:0.789–0.895)	0.727 (95%CI:0.677–0.760)
GBM	0.837 (95%CI:0.777–0.896)	0.705 (95%CI:0.683–0.749)
KNN	0.864 (95%CI:0.835–0.893)	0.709 (95%CI:0.672–0.831)
Stacking	0.959 (95%CI:0.942–0.977)	0.815 (95%CI:0.794–0.843)

**Figure 8:**
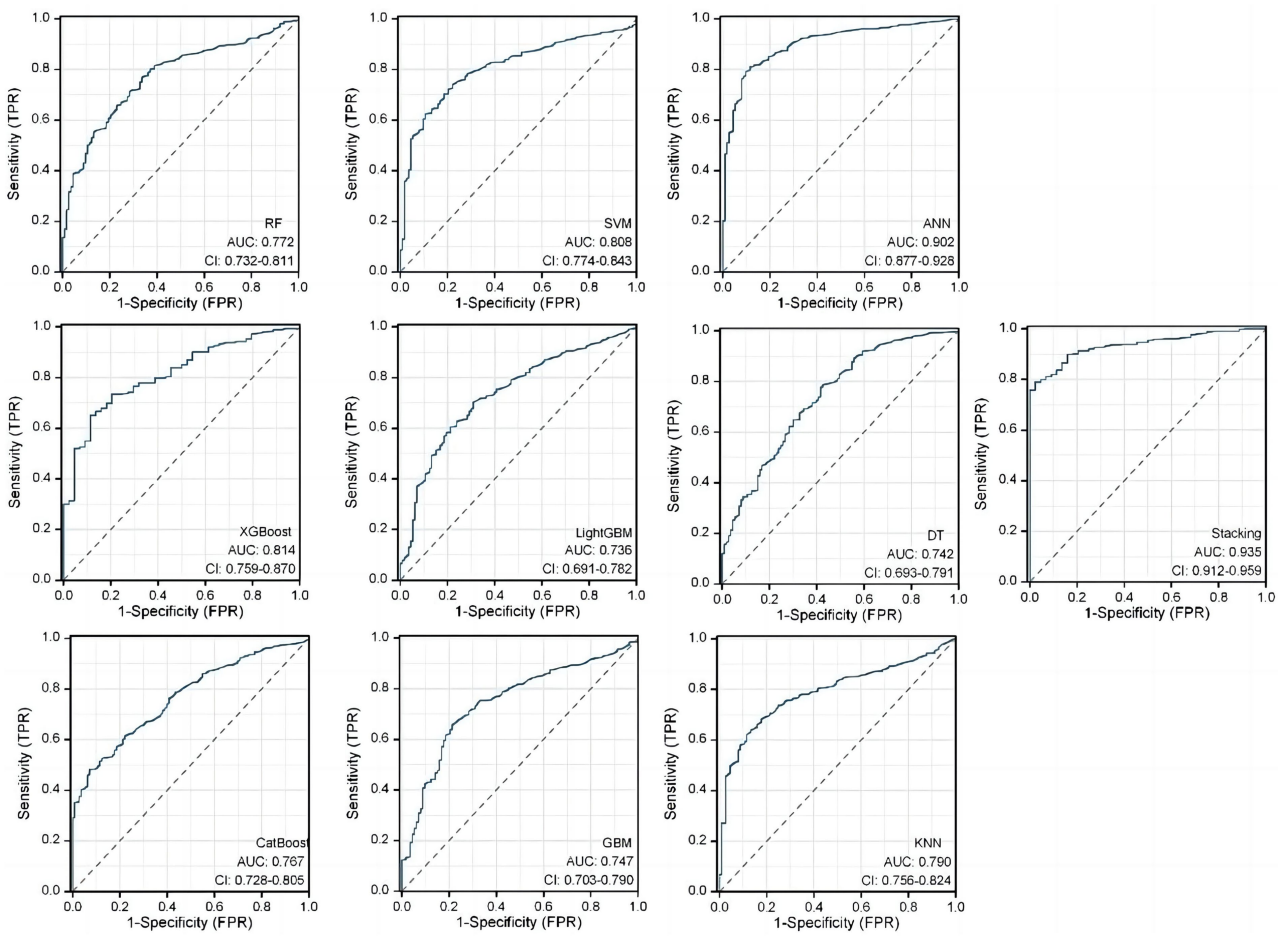
Predictive ability of the 10 RSMs evaluated using the AUC of the ROC curve.

**Figure 9:**
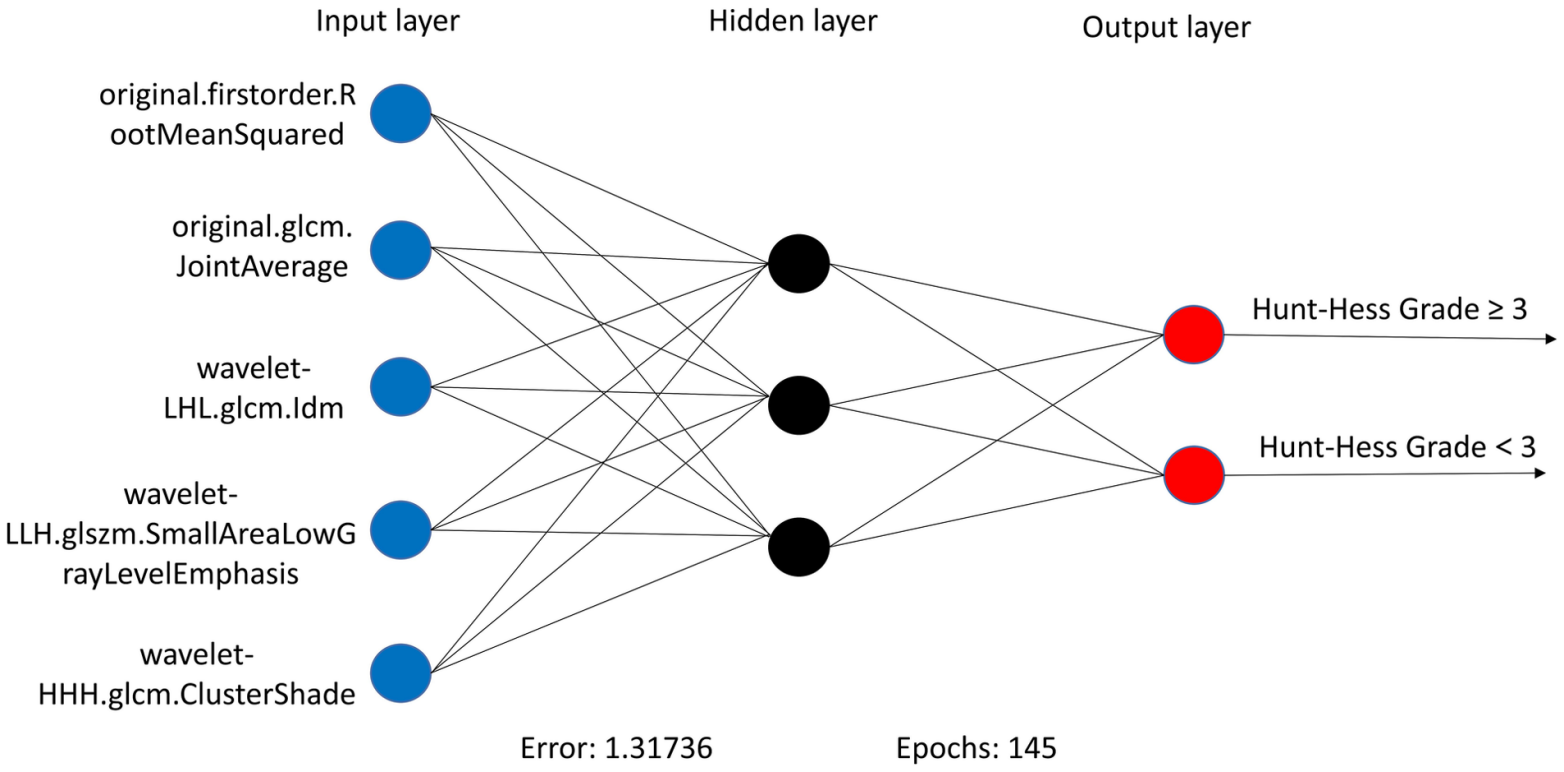
ANN algorithms for constructing RSM visualization, consisting of five input layers, three hidden layers, and two output layers.

In the RSMs category, the ANN continued to show favorable performance. The AUC of the ANN model for the RSM is 0.902 and the MCC is 0.802, which are better than the other base models (Table 5 and Figure 8). The ANN model used to construct the RSM was trained with 145 epochs to achieve a minimum error rate of 1.31736 (Figure 9).

**Table 5:** Predictive ability of the 10 RSMs evaluated using the AUC and MCC.

Model	AUC	MCC
RF	0.772 (95%CI:0.732–0.811)	0.635 (95%CI:0.601–0.694)
SVM	0.808 (95%CI:0.774–0.843)	0.694 (95%CI:0.635–0.730)
ANN	0.902 (95%CI:0.877–0.928)	0.802 (95%CI:0.770–0.838)
XGBoost	0.814 (95%CI:0.759–0.870)	0.700 (95%CI:0.671–0.766)
LightGBM	0.736 (95%CI:0.691–0.782)	0.608 (95%CI:0.552–0.649)
DT	0.742 (95%CI:0.693–0.791)	0.634 (95%CI:0.605–0.676)
CatBoost	0.767 (95%CI:0.728–0.805)	0.628 (95%CI:0.599–0.663)
GBM	0.747 (95%CI:0.703–0.790)	0.657 (95%CI:0.618–0.690)
KNN	0.790 (95%CI:0.756–0.824)	0.691 (95%CI:0.628–0.731)
Stacking	0.935 (95%CI:0.912–0.959)	0.793 (95%CI:0.760–0.832)

**Figure 10:**
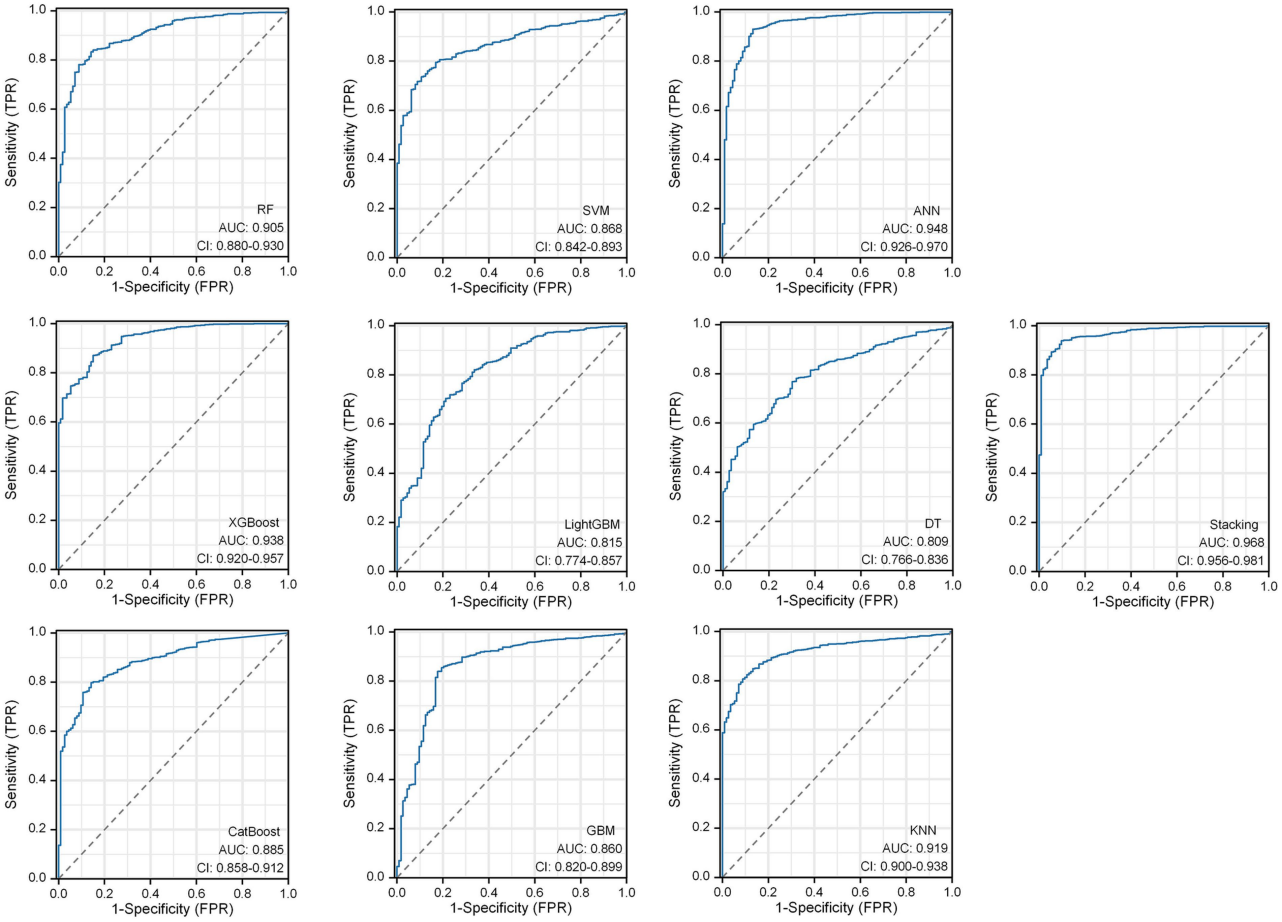
Predictive ability of 10 DLRSCMs evaluated using the AUC of ROC curves.

However, the most notable performance was observed in the DLRSCMs category. The Stacking ensemble model, which incorporated ANN as its secondary learner due to its superior performance among the basic models, achieved an AUC of 0.968 and an MCC of 0.820 (Table 6 and Figure 10). This high level of performance reflects the efficacy of combining various predictive models, particularly leveraging the strengths of the best-performing base model, in this case, the ANN, to enhance the overall predictive accuracy and reliability of the ensemble model. This approach underlines the potential of utilizing sophisticated model integration strategies, like Stacking ensemble models, in machine learning tasks to achieve optimal results.

**Table 6:** Predictive ability of the 10 DLRSCMs, evaluated using the AUC and MCC.

Model	AUC	MCC
RF	0.905 (95%CI:0.880–0.930)	0.785 (95%CI:0.752–0.810)
SVM	0.868 (95%CI:0.842–0.893)	0.729 (95%CI:0.698–0.756)
ANN	0.948 (95%CI:0.926–0.970)	0.804 (95%CI:0.765–0.842)
XGBoost	0.938 (95%CI:0.920–0957)	0.800 (95%CI:0.762–0.831)
LightGBM	0.815 (95%CI:0.774–0.857)	0.701 (95%CI:0.668–0.737)
DT	0.809 (95%CI:0.766–0.836)	0.682 (95%CI:0.659–0.722)
CatBoost	0.885 (95%CI:0.858–0.912)	0.707 (95%CI:0.675–0.734)
GBM	0.860 (95%CI:0.820–0.899)	0.703 (95%CI:0.681–0.739)
KNN	0.919 (95%CI:0.900–0.938)	0.795 (95%CI:0.753–0.838)
Stacking	0.968 (95%CI:0.956–0.981)	0.820 (95%CI:0.788–0.851)

#### 3.4.4 Construction of rad-scores and nomogram model

We constructed a Rad-Score based on the five radiomics features screened after Lasso regression, which was calculated as follows:


$$\begin{array}{l} \mathrm{Radiomics}\;\mathrm{Score}=\overset{n}{\underset{i=1}{\sum }}\left(\beta_{i}\times X_{i}\right)=0.000922077313959615\\ \;*\mathrm{Feature}1+0.0785993197007064\\ \;*\mathrm{Feature}2+0.701727629619912\\ \;*\mathrm{Feature}3-8.4144084384922\\ \;*\mathrm{Feature}4-0.0361369923060118*\mathrm{Feature}5 \end{array}$$

**Figure 11:**
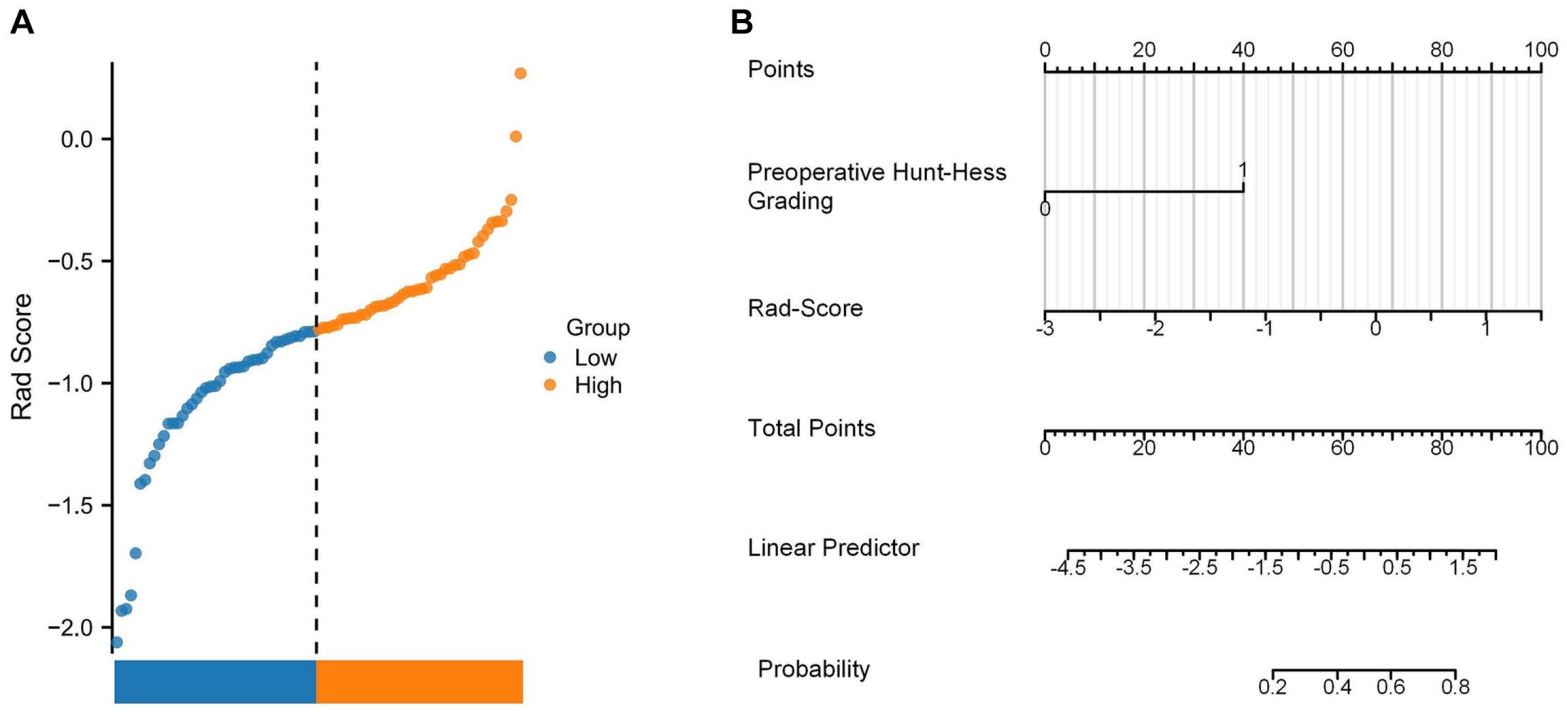
**(A)** Rad-Score distribution plot for all patients. **(B)** Nomogram constructed using the combination of Rad-Score and preoperative Hunt–Hess grading.

**Figure 12:**
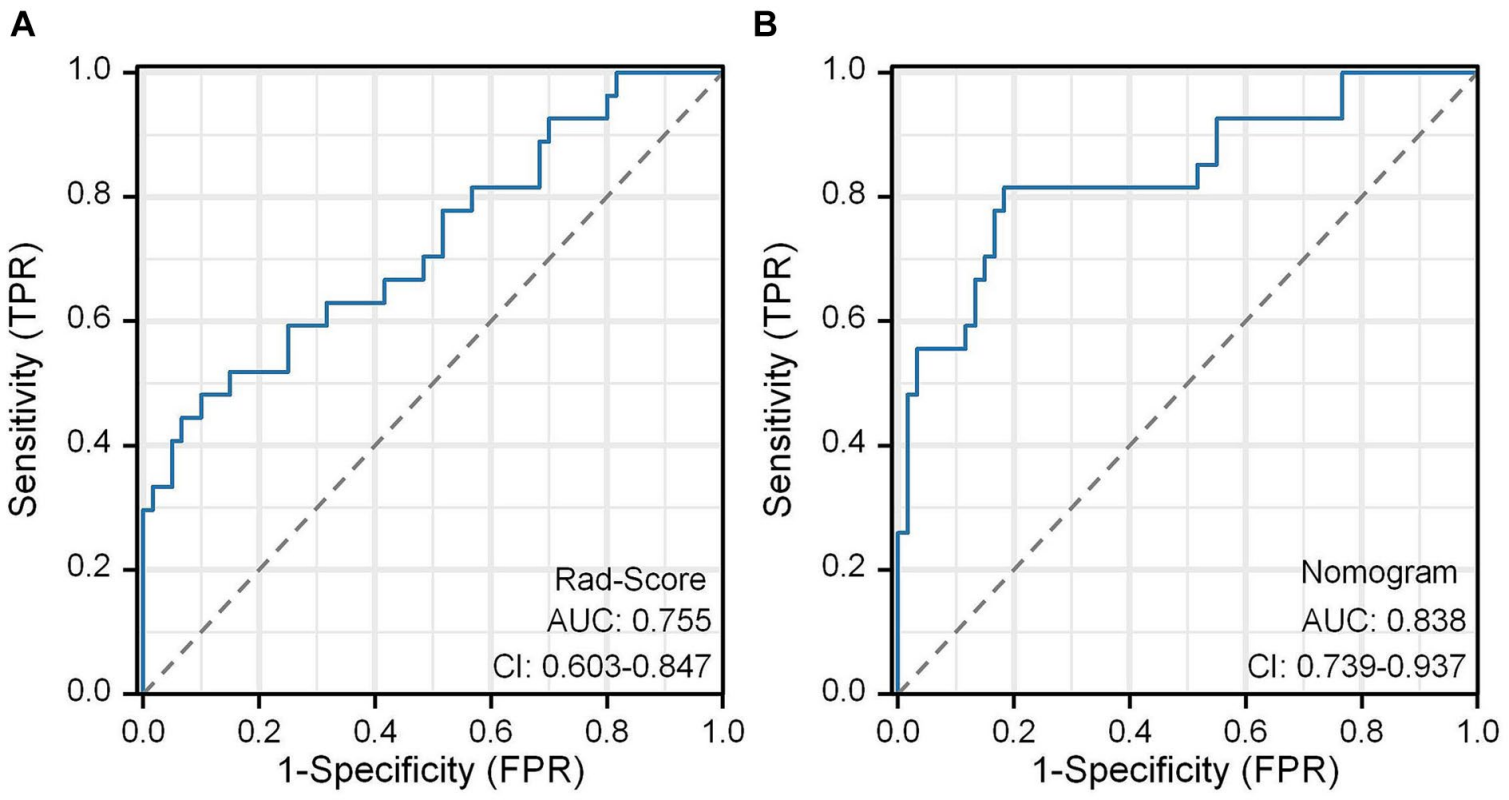
ROC curves of the Rad-Score model **(A)** and the nomogram model **(B)**.

In this study, the Rad-Score of all patients in the dataset was calculated and the distribution of patients was plotted according to the above equation (Figure 11A). The univariate regression analysis showed that preoperative Hunt-Hess grading was an independent predictor. Therefore, we combined Rad-Score and preoperative Hunt-Hess grading to construct the Nomogram (Figure 11B). The predictive performance of Rad-Score and Nomogram, respectively, was internally validated using 1,000 resamplings using the Bootstrap method, and the results showed that the AUC of Rad-Score was 0.755 (95% CI:0.603–0.847) (Figure 12A). The AUC of Nomogram was 0.838 (95% CI:0.739–0.937) (Figure 12B). The nomogram combining Rad-Score and preoperative Hunt–Hess grading demonstrated improved predictive ability compared with a single predictor.

#### 3.4.5 Further exploration of the model performance

**Figure 13:**
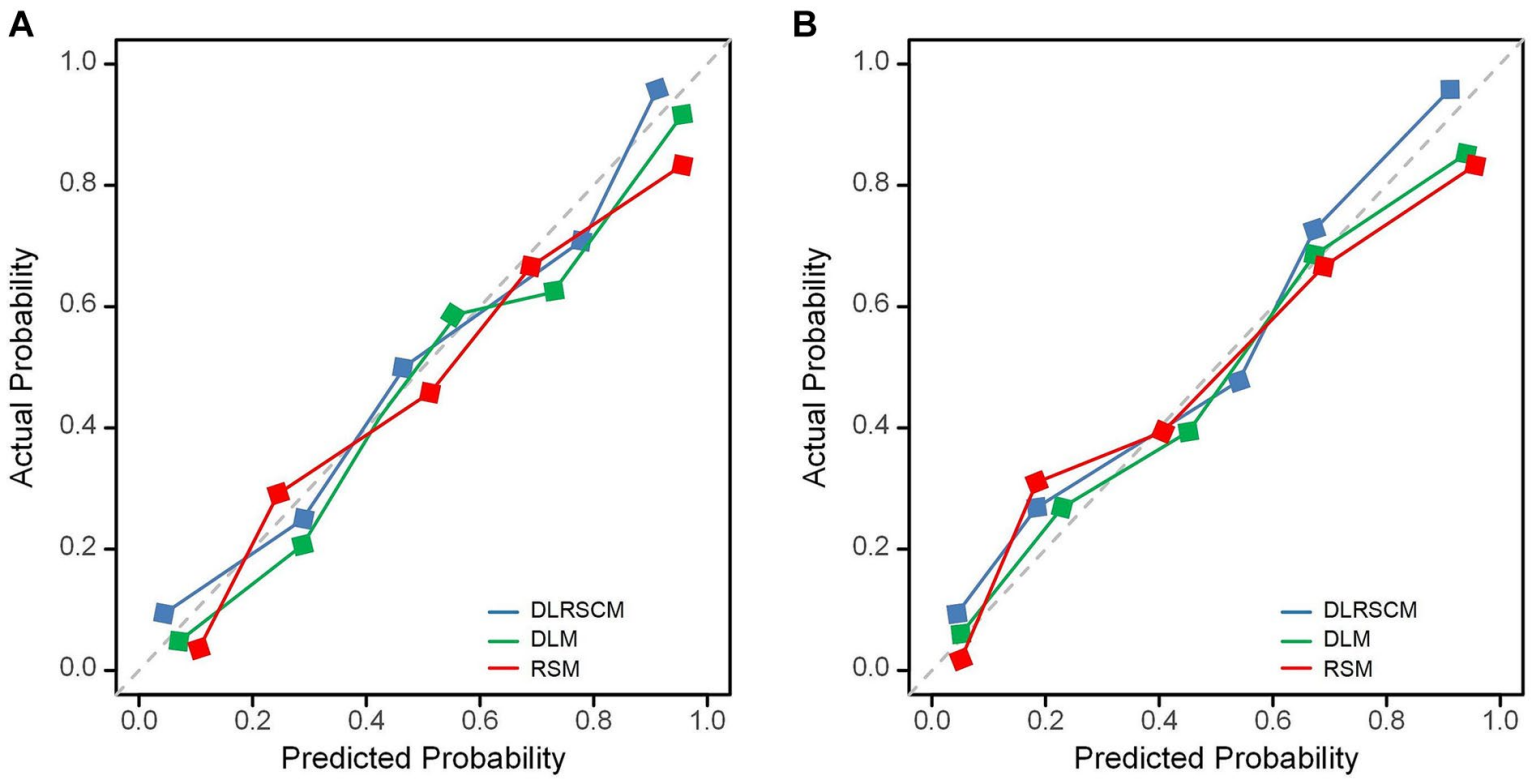
Calibration curves for predictive models. Calibration curves of three types of models constructed using ANN algorithms **(A)** and stacking **(B)**. DLM, Deep learning feature-based model; DLRSCM, deep learning–radiomics fusion prediction model; RSM, radiomics model.

**Figure 14:**
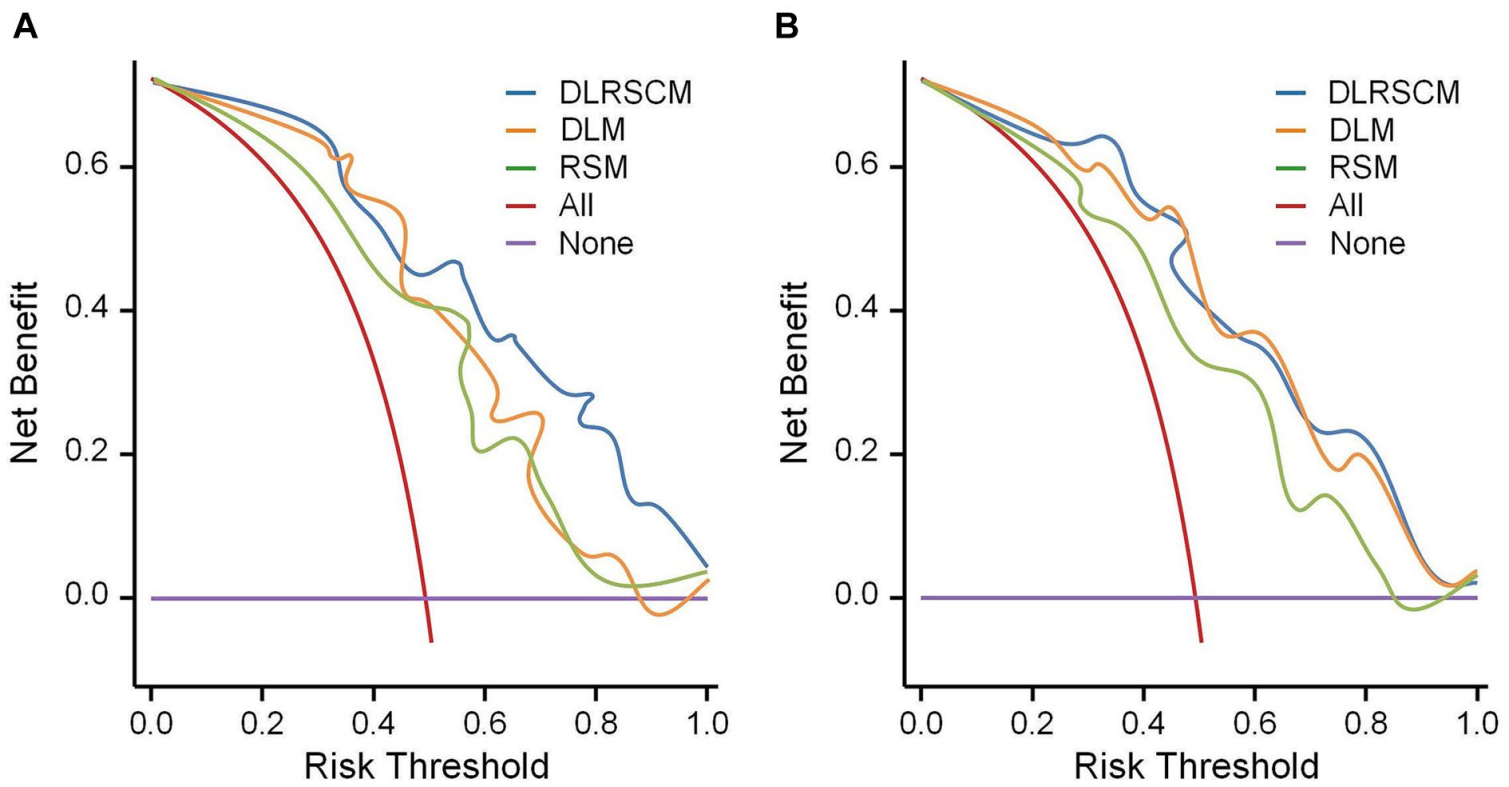
Decision curve analysis for the prediction model. Decision curve analysis of three types of models constructed using ANN algorithms **(A)** and stacking **(B)**. DLM, Deep learning feature-based model; DLRSCM, deep learning–radiomics fusion prediction model; RSM, radiomics model.

In this study, we used both calibration curve and decision curve analysis (DCA) to evaluate the performance of the stacking and ANN models for each type of model. We assessed the accuracy and reliability of the model predictions using the calibration curve, whereas DCA helped comprehensively evaluate the clinical application of the model under different patient risk thresholds. Using calibration curves, we found that all ANN and stacking model predictions were in good agreement with the actual observations predicting the Hunt–Hess grading (Figures 13A,B). DCA showed that DLRSCM had a better clinical net benefit compared with the other single-scale models in the ANN-constructed basic model (Figure 14A). However, DLM and DLRSCM had similar clinical net benefits in the stacking model (Figure 14B). Overall, the stacking model had better clinical net benefits than the ANN basic model.

## 4 Discussion

The three categories of Stacking ensemble models constructed in this study exhibited good performance in predicting postoperative Hunt-Hess grading. The highest model performance was observed when deep learning features were fused with radiomics features, achieving an AUC of 0.968 (95% CI = 0.956–0.981).

Zhang et al. (37) identified several laboratory test indicators, such as the C-reactive protein to lymphocyte ratio (AUC = 0.840), through Multivariate logistic regression analysis. They determined that C-reactive protein is a preoperative predictor of postoperative Hunt-Hess grading (AUC = 0.838). In another study, Zheng et al. (38) used preoperative serum lactate dehydrogenase levels as an independent predictor for postoperative Hunt-Hess grading. They found that preoperative serum lactate dehydrogenase levels were a risk factor for postoperative neurological function (AUC = 0.702) and were associated with limited outcomes. Compared to these studies, our fusion model, which integrates significant radiomics features and deep learning features and is trained through the Stacking ensemble algorithm, demonstrates superior predictive performance (AUC = 0.968). Additionally, decision curve analysis indicates that the fusion model has a higher clinical net benefit compared to models using only radiomics features or deep learning features alone in the current internal validation. This suggests that the fusion model may have potential clinical utility.

In previous studies, preoperative Hunt-Hess grading has been shown to have significant correlations with various adverse outcomes of subarachnoid hemorrhage (5). In our study, we also found a significant correlation between preoperative and postoperative Hunt-Hess grading (*p* < 0.05), corroborating previous findings that preoperative Hunt-Hess grading can significantly influence postoperative neurological function (38). A Nomogram combining preoperative Hunt-Hess grading with Rad-Score showed better predictive ability for postoperative Hunt-Hess grading (AUC = 0.838) compared to several individual models across three categories. However, the predictive performance of the Nomogram still has a gap to Stacking ensemble model in each category.

While the Stacking ensemble models across different categories outperformed the Nomogram model, the Nomogram offers simplicity and more direct patient outcome assessment, making it more interpretable. Therefore, future research on predictive models should focus more on the interpretability of the models. In the performance comparison across model categories, the Deep Learning-Radiomics Signature Combined Model (DLRSCM) showed better overall performance than the Deep Learning Model (DLM), which in turn outperformed the Radiomics Signature Model (RSM). This difference in performance might be attributed to the DLRSCM model’s fusion of radiomics features and deep learning features, providing a more comprehensive data perspective. This integrated approach can capture more data patterns and subtle differences that might be overlooked in single-source feature models, such as those using only RSM or DLM. The advantage of DLRSCM lies in its combination of the strengths of both types of data: radiomics features provide intuitive, interpretable medical imaging information, while deep learning features extract deeper and more abstract patterns from these images. Thus, DLRSCM is more effective in capturing complex patterns associated with postoperative Hunt-Hess grading.

The ANN displayed excellent prediction performance among all three models in the basic model. This might be related to the algorithmic structure of ANN itself. The superior performance of ANN among the three basic models could be attributed to its ability to handle complex nonlinear relationships, adaptive learning capability, advantages in processing large-scale data, advanced feature extraction ability, and flexibility and generalization power (39). First, as a robust nonlinear model, ANN could effectively process datasets with intricate nonlinear features, making it advantageous in capturing diverse and abstract characteristics, particularly in medical imaging domains (40). Second, the adaptive learning capacity of ANN, achieved using the backpropagation algorithm, optimized model parameters, gradually adapting to data features and enhancing predictive accuracy (41). Additionally, ANN excelled in processing large-scale data, especially in deep learning, where its multi-layered network structure efficiently handled high-dimensional complex data, thereby bolstering model performance (42). Furthermore, the ability of ANN to automatically learn advanced feature representations enabled the mining of more comprehensive data information compared with traditional feature extraction methods, leading to improved predictive outcomes (43).

The stacking ensemble model demonstrated surprisingly outstanding performance in our study. Stacking is an ensemble learning method that combines predictions from multiple base models to train a meta-model for final prediction. In our study, the stacking models were constructed for each type of model used. We selected diverse base models constructed using different algorithms. We leveraged the strengths of each model while compensating for their individual weaknesses by incorporating the predictions of these base models into the stacking ensemble (44). The stacking model learned the relationships between predictions from different base models, leading to further improvement in predictive performance. Its learning capability allowed for the weighted combination of predictions from different models, resulting in more accurate and robust predictions (45).

This study also has some limitations. Firstly, the small sample size and data collection from a single center may limit the generalizability of the findings. The absence of external validation is another significant limitation, as it is crucial for confirming the efficacy and robustness of our models. Finally, the dependency of radiomics and deep learning models on imaging quality means that variations in imaging protocols and equipment could impact the models’ effectiveness across different clinical settings.

The state-of-the-art machine learning and deep learning technologies, such as Transformers, hold immense potential in clinical predictive modeling. Rao et al. (46) have utilized Transformers to construct a predictive model for heart failure events, which not only demonstrates strong predictive performance but also provides insights for data-driven risk factor identification. Transformers offer a potential direction for our future research. However, large model architectures like Transformers rely on training with large datasets. Current studies based on Transformers have used over one hundred thousand samples for model training (46–48). Therefore, due to the small sample size in our study, we did not adopt this model methodology.

In our future work, we plan to address these limitations by expanding the scope of our research to encompass larger and more diverse patient populations. This expansion will significantly enhance the robustness and applicability of our research findings. In conjunction with this expansion, we also aim to explore the integration of advanced machine learning technologies such as Transformers into our predictive modeling framework. Our goal is to develop more sophisticated and accurate predictive tools that can be effectively applied in clinical settings, ultimately contributing to improved patient care.

## 5 Conclusion

This study findings might significantly affect clinical practice in managing IAs. The accurate prediction of postoperative Hunt–Hess classification plays a reference in clinical practice. It enables clinicians to perform risk stratification and develop personalized treatment plans. Early identification of patients at higher risk of neurological complications allows for timely interventions, potentially reducing the burden of postoperative complications and improving patient outcomes.

## Data availability statement

The raw data supporting the conclusions of this article will be made available by the authors, without undue reservation.

## Ethics statement

The studies involving humans were approved by Medical Ethics Committee of the Affiliated Hospital of Southwest Medical University. The studies were conducted in accordance with the local legislation and institutional requirements. The ethics committee/institutional review board waived the requirement of written informed consent for participation from the participants or the participants’ legal guardians/next of kin because the requirement for obtaining informed consent from the patients was waived due to the retrospective nature of this study.

## Author contributions

YP: Conceptualization, Data curation, Formal analysis, Funding acquisition, Investigation, Methodology, Resources, Software, Validation, Visualization, Writing – original draft, Writing – review & editing. YW: Conceptualization, Data curation, Formal analysis, Funding acquisition, Investigation, Methodology, Resources, Software, Validation, Visualization, Writing – original draft, Writing – review & editing. ZW: Data curation, Formal analysis, Investigation, Methodology, Resources, Software, Validation, Writing – original draft. HX: Data curation, Formal analysis, Investigation, Methodology, Validation, Visualization, Writing – original draft. LG: Data curation, Formal analysis, Methodology, Resources, Software, Writing – original draft. LS: Data curation, Investigation, Resources, Software, Writing – original draft. YH: Data curation, Software, Visualization, Writing – original draft. HP: Conceptualization, Data curation, Funding acquisition, Project administration, Supervision, Validation, Writing – review & editing. PZ: Project administration, Supervision, Validation, Writing – review & editing, Conceptualization, Data curation, Funding acquisition. XZ: Conceptualization, Data curation, Funding acquisition, Project administration, Resources, Supervision, Visualization, Writing – original draft, Writing – review & editing.
