# Supplementary material for: Deep learning and machine learning predictive models for neurological function after interventional embolization of intracranial aneurysms
Source: Front Neurol. 2024 Jan 24;15:1321923. doi: 10.3389/fneur.2024.1321923 (PMC10848172; doi:10.3389/fneur.2024.1321923)
Supplement: Supplementary file 1 [file Table_1.DOCX]

**Supplementary Files：**

**Radiomics Feature-based Model**

RF (n_estimators=1000, max_depth=17, min_samples_split=10)

SVM (C=0.8, kernel=linear)

ANN (hidden_layer_sizes=3, activation=relu, solver=adam, learning_rate_init=10^-4^, max_iter=500)

XGBoost (n_estimators=1000, eta=0.01, max_depth=5, subsample=0.7, colsample_bytree=0.4)

LightGBM (num_leaves=54, learning_rate=0.05, n_estimators=800, max_depth=4)

DT (max_depth=10, min_samples_split=9, min_samples_leaf=7)

CatBoost (iterations=1000, learning_rate=0.01, depth=7)

GBM (n_estimators=500, learning_rate=0.01, max_depth=6)

KNN (n_neighbors=8)

**Deep Learning Feature-based Model**

RF (n_estimators=1000, max_depth=18, min_samples_split=13)

SVM (C=0.7, kernel=linear)

ANN (hidden_layer_sizes=2, activation=relu, solver=adam, learning_rate_init=10^-4^, max_iter=500)

XGBoost (n_estimators=1000, eta=0.01, max_depth=6, subsample=0.7, colsample_bytree=0.5)

LightGBM (num_leaves=41, learning_rate=0.03, n_estimators=800, max_depth=3)

DT (max_depth=7, min_samples_split=8, min_samples_leaf=6)

CatBoost (iterations=1000, learning_rate=0.02, depth=6)

GBM (n_estimators=500, learning_rate=0.02, max_depth=5)

KNN (n_neighbors=6)

**Deep Learning-Radiomics Feature Fusion model**

RF (n_estimators=1000, max_depth=15, min_samples_split=12)

SVM (C=1, kernel=linear)

ANN (hidden_layer_sizes=7, activation=relu, solver=adam, learning_rate_init=10^-4^, max_iter=500)

XGBoost (n_estimators=1000, eta=0.01, max_depth=8, subsample=0.8, colsample_bytree=0.3)

LightGBM (num_leaves=78, learning_rate=0.05, n_estimators=1000, max_depth=7)

DT (max_depth=14, min_samples_split=5, min_samples_leaf=9)

CatBoost (iterations=1000, learning_rate=0.01, depth=8)

GBM (n_estimators=500, learning_rate=0.01, max_depth=8)

KNN (n_neighbors=9)
